# Supplementary material for: Mechanisms governing the pioneering and redistribution capabilities of the non-classical pioneer PU.1
Source: Nat Commun. 2020 Jan 21;11:402. doi: 10.1038/s41467-019-13960-2 (PMC6972792; doi:10.1038/s41467-019-13960-2)
Supplement: Supplementary file 7 — Source data [file 41467_2019_13960_MOESM7_ESM.zip › Source_Data/FigureS2/FigureS2B_MotifScanOutput/KmeansCluster4/homerResults/motif11.similar.html]

motif11

## Information for motif11

C
T
G
A
T
C
G
A
T
G
C
A
C
T
G
A
T
G
C
A
C
G
T
A
C
A
T
G
C
A
T
G
T
C
A
G
T
G
C
A
G
C
T
A
T
G
A
C
G
A
C
T
  
Reverse Opposite:  

C
T
G
A
A
C
T
G
C
G
A
T
A
C
G
T
A
G
T
C
G
T
A
C
G
T
A
C
G
C
A
T
A
C
G
T
G
A
C
T
A
C
G
T
A
G
C
T
G
A
C
T
  

|  |  |
| --- | --- |
| p-value: | 1e-79 |
| log p-value: | -1.825e+02 |
| Information Content per bp: | 1.689 |
| Number of Target Sequences with motif | 290.0 |
| Percentage of Target Sequences with motif | 6.42% |
| Number of Background Sequences with motif | 603.3 |
| Percentage of Background Sequences with motif | 1.44% |
| Average Position of motif in Targets | 102.0 +/- 46.7bp |
| Average Position of motif in Background | 100.9 +/- 72.2bp |
| Strand Bias (log2 ratio + to - strand density) | -0.1 |
| Multiplicity (# of sites on avg that occur together) | 1.05 |
| Motif File: | file (matrix) reverse opposite |

### Similar de novo motifs found

|  |  |  |  |  |  |  |  |
| --- | --- | --- | --- | --- | --- | --- | --- |
| Rank | Match Score | Redundant Motif | P-value | log P-value | % of Targets | % of Background | Motif file |
| 1 | 0.794 | C T G A C G T A C T G A G A T C C T A G A C T G T A C G C G T A C G T A G T A C T G A C G A T C | 1e-28 | -64.695046 | 1.44% | 0.18% | motif file (matrix) |
